# Supplementary figures and images for: Laparoscopic Peritoneal Wash Cytology-Derived Primary Human Mesothelial Cells for In Vitro Cell Culture and Simulation of Human Peritoneum
Source: Biomedicines. 2021 Feb 10;9(2):176. doi: 10.3390/biomedicines9020176 (PMC7916778; doi:10.3390/biomedicines9020176)

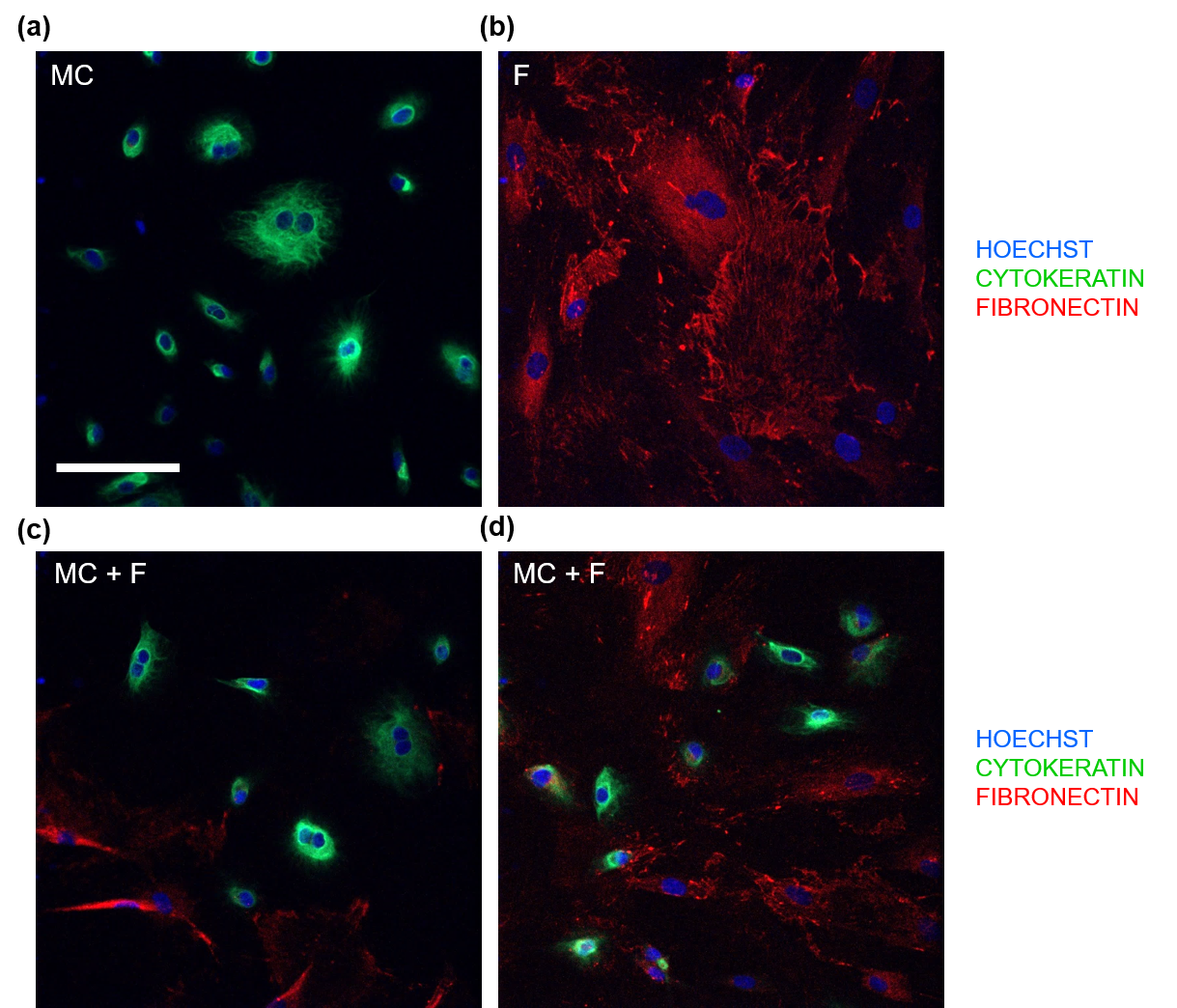

Supplement: Supplementary file 1 [file biomedicines-09-00176-s001.zip › Figure S1.tif]

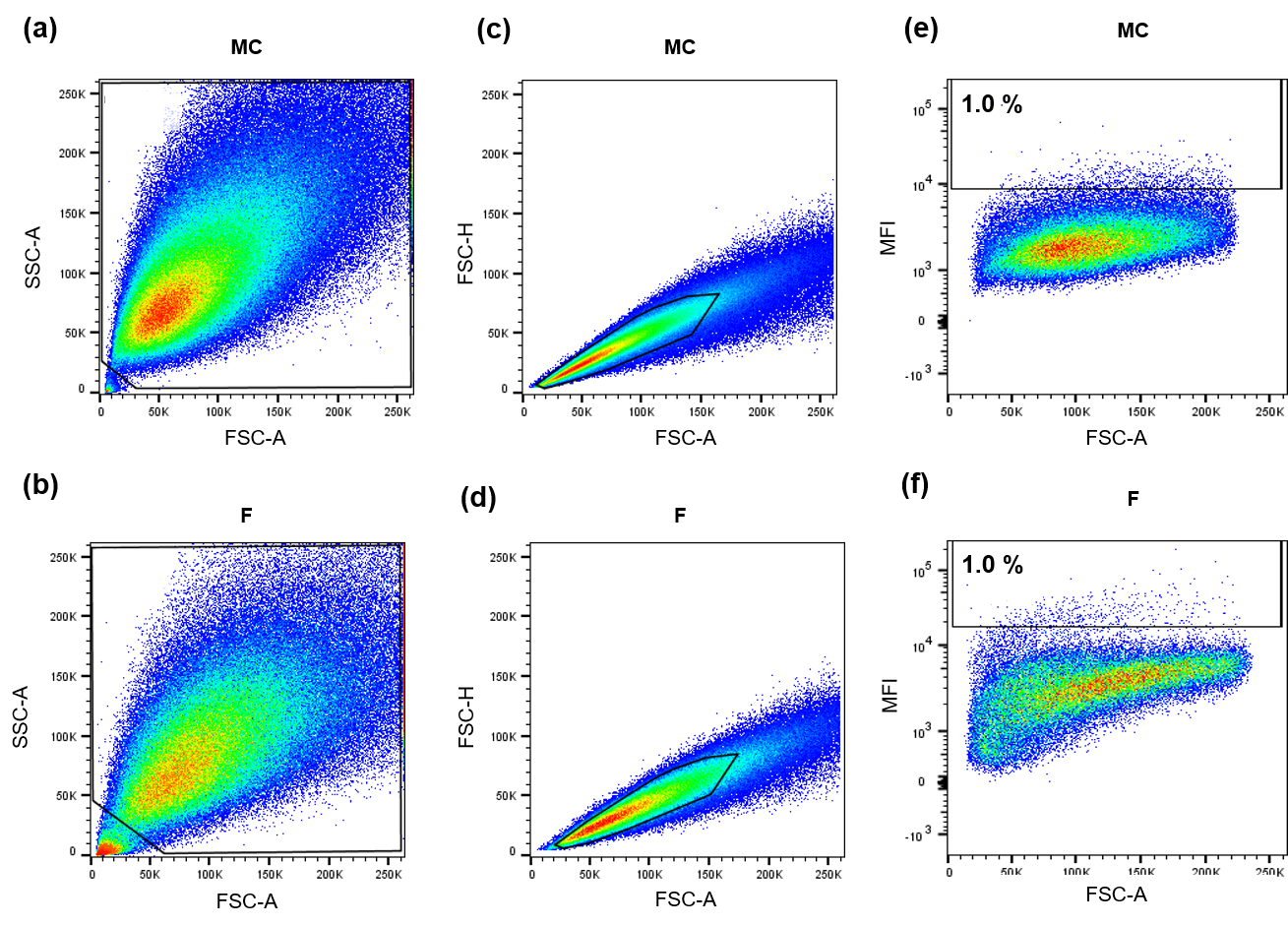

Supplement: Supplementary file 1 [file biomedicines-09-00176-s001.zip › Figure S2.tif]

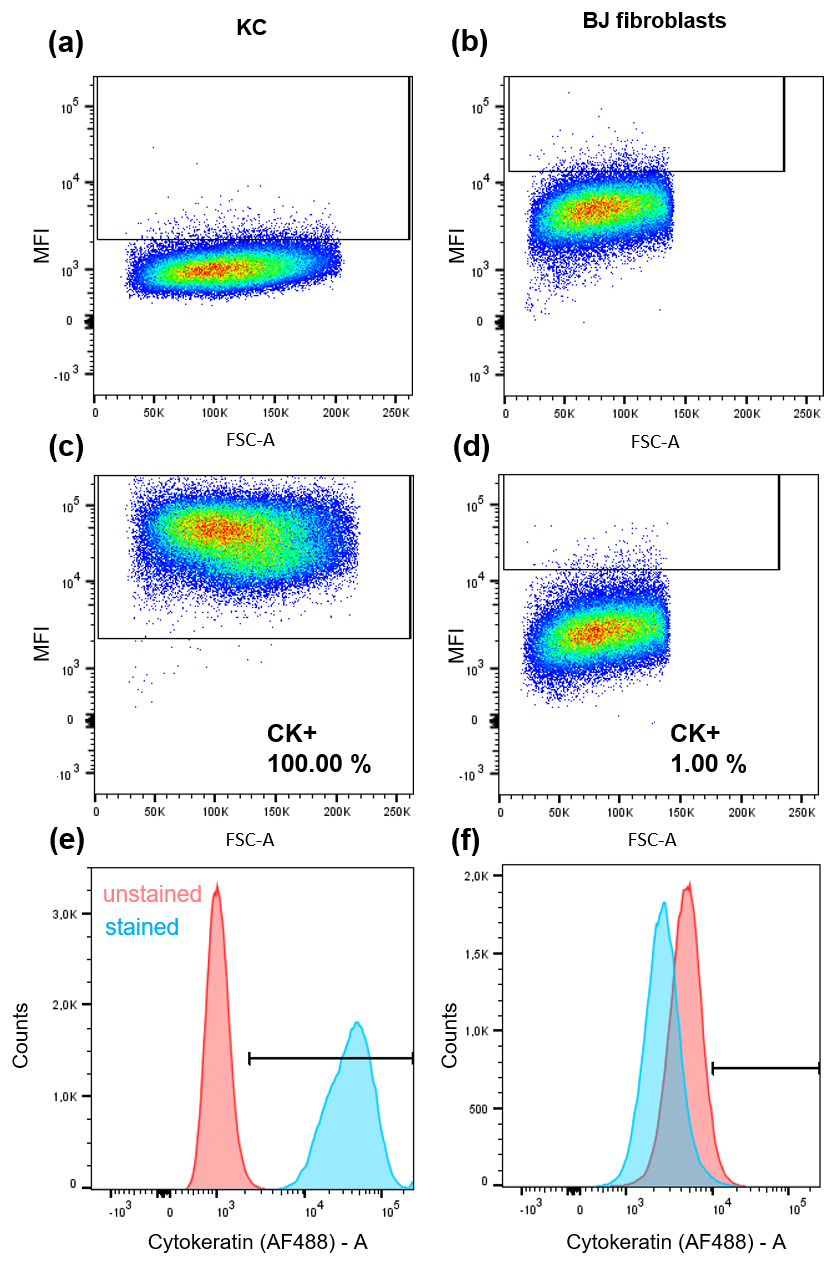

Supplement: Supplementary file 1 [file biomedicines-09-00176-s001.zip › Figure S3.tif]
